# Genetics of Na^+^ exclusion and salinity tolerance in Afghani durum wheat landraces

**DOI:** 10.1186/s12870-017-1164-6

**Published:** 2017-11-21

**Authors:** Nawar Jalal Shamaya, Yuri Shavrukov, Peter Langridge, Stuart John Roy, Mark Tester

**Affiliations:** 1 Department of Field Crops, College of Agriculture, University of Baghdad, Al-Jadriyah, Baghdad, Iraq; 2 School of Agriculture, Food and Wine, The University of Adelaide, Urrbrae, SA 5064 Australia; 3 Australian Centre for Plant Functional Genomics, The University of Adelaide, Urrbrae, SA 5064 Australia; 4 Center for Desert Agriculture, Division of Biological and Environmental Sciences and Engineering, King Abdullah University of Science and Technology, Thuwal, 4700 Kingdom of Saudi Arabia

**Keywords:** Salinity tolerance, Leaf Na^+^ concentration, K^+^/Na^+^ ratio, Bulk Segregant analysis, SNP markers, *HKT1;5*

## Abstract

**Background:**

Selecting for low concentration of Na^+^ in the shoot provides one approach for tackling salinity stress that adversely affects crop production. Novel alleles for Na^+^ exclusion can be identified and then introduced into elite crop cultivars.

**Results:**

We have identified loci associated with lower Na^+^ concentration in leaves of durum wheat landraces originating from Afghanistan. Seedlings of two F_2_ populations derived from crossings between Australian durum wheat (Jandaroi) and two Afghani landraces (AUS-14740 and AUS-14752) were grown hydroponically and evaluated for Na^+^ and K^+^ concentration in the third leaf. High heritability was found for both third leaf Na^+^ concentration and the K^+^/Na^+^ ratio in both populations. Further work focussed on line AUS-14740. Bulk segregant analysis using 9 K SNP markers identified two loci significantly associated with third leaf Na^+^ concentration. Marker regression analysis showed a strong association between all traits studied and a favourable allele originating from AUS-14740 located on the long arm of chromosome 4B.

**Conclusions:**

The candidate gene in the relevant region of chromosome 4B is likely to be the high affinity K^+^ transporter B1 (*HKT1;5-B1*). A second locus associated with third leaf Na^+^ concentration was located on chromosome 3BL, with the favourable allele originating from Jandaroi; however, no candidate gene can be identified.

**Electronic supplementary material:**

The online version of this article (10.1186/s12870-017-1164-6) contains supplementary material, which is available to authorized users.

## Background

Plants vary widely in tolerance to salt stress, with durum wheat being particularly sensitive [1]. Commercial durum wheat is grown in temperate regions of the world, under both irrigated and rain-fed systems [2]. Much of the world’s arable land, however, is affected by salinity, and this is a major limitation for durum wheat production [3, 4]. Low shoot Na^+^ concentration is necessary for normal growth of most cereals. Indeed, maintenance of a high K^+^/Na^+^ ratio in the shoot was identified as one of the major mechanisms associated with salinity tolerance in the *Triticeae* and other plants [5]. Tetraploid durum wheat cultivars (*Triticum turgidum ssp. durum*) are more sensitive to salinity than hexaploid bread wheat (*T. aestivum*) and this is associated with their high concentration of Na^+^ in the shoots [3, 4].

Several attempts have been made to improve Na^+^ exclusion and the ratio of K^+^ / Na^+^ in durum wheat. Natural variability for salinity tolerance among durum wheat cultivars is very limited [6] and this can be increased by introducing traits from the D-genome of bread wheat or through the use of landraces or wild species [7]. The *Kna1* locus on the long arm of chromosome 4D in bread wheat, found to be responsible for maintaining beneficial K^+^/Na^+^ ratios, was introgressed into the long arm of chromosome 4B in durum wheat using disomic substitution lines [7]. Although a novel durum line with lower shoot Na^+^ concentration and a higher shoot K^+^/Na^+^ ratio was generated, this line produced a lower biomass compared with other durum wheat cultivars. It was later found that *Kna1* was also linked with undesirable traits [8].

Another approach to enhance Na^+^ exclusion in durum wheat involves the screening of large numbers of accessions and landraces to identify new sources of Na^+^ exclusion which can be introduced into elite durum cultivars. Wild relatives of wheat have been used for many years in breeding programmes as a source for novel alleles and traits for improving yield under abiotic and biotic stress. In the area of salinity research, the Indian landrace Kharchia 65 was found to have high exclusion of Na^+^ from the leaves [9] and has been widely used to develop salt tolerant breeding lines and commercial cultivars of bread wheat [10]. Bread wheat landraces from Iran, Nepal and Pakistan have been found to contain novel sources of salinity tolerance which have been used to improve yield in saline fields [11, 12].

However, durum wheat landraces have only rarely been used for the improvement of salinity tolerance. A source of Na^+^ exclusion was identified in durum wheat line 149, which was derived from a cross between a wild diploid species *T. monococcum*, accession C68–101, and the durum wheat cv. Marrocos [6]. The shoot Na^+^ exclusion phenotype was associated with two loci, one on the long arm of chromosome 2A (*Nax1*) [13] and the second on the long arm of chromosome 5A (*Nax2*) [14]. These loci were the site of an old translocation from *T. monococcum* [15]. Further investigation revealed that the *Nax1* locus is associated with Na^+^ exclusion by unloading Na^+^ in the leaf sheath so reducing leaf blade Na^+^ and increasing leaf blade K^+^. This locus is also associated with the retrieval of Na^+^ ions from the xylem in the roots before they can be transported to the shoots. The second locus, *Nax2*, is also associated with the retrieval of Na^+^ from the xylem, although only in the roots, before the ions can be transported to the shoots [14]. The candidate genes for the *Nax1* and *Nax2* loci were identified as *TmHKT1;4-A* and *TmHKT1:5-A*, respectively. *TmHKT1;5-A*, is a homoeolog to the bread wheat gene, *TaHKT1;5-D*, which is the candidate gene for the *Kna1* locus [15, 16]. The introgression of *TmHKT1;5* (*Nax2*) into near-isogenic tetraploid lines with a cv. Tamaroi background resulted in a yield enhancement of 26% under salinized field conditions [17].

In earlier work, two Afghani durum landraces, Gandum Siahloshe Zamistani Aubi (AUS-14740) and Gandum Kofari (AUS-14752), were found to accumulate approximately half the concentration of Na^+^ in the third leaf compared with the other 177 durum wheat cultivars and landraces screened [18]. Despite their common geographical origin, lines AUS-14740 and AUS-14752 had different growth habits and plant height, corresponding presumably to different genetic backgrounds. Therefore, it is possible that lines AUS-14740 and AUS-14752 may be sources of novel alleles of genes controlling Na^+^ exclusion and a high K^+^/Na^+^ ratio. The aim of the present study was to determine the genetic control of Na^+^ exclusion and the K^+^/Na^+^ ratio in segregating populations of durum wheat derived from these two exotic Afghani landraces.

## Results

### Phenotyping and types of genetic segregations

**Fig. 1:**
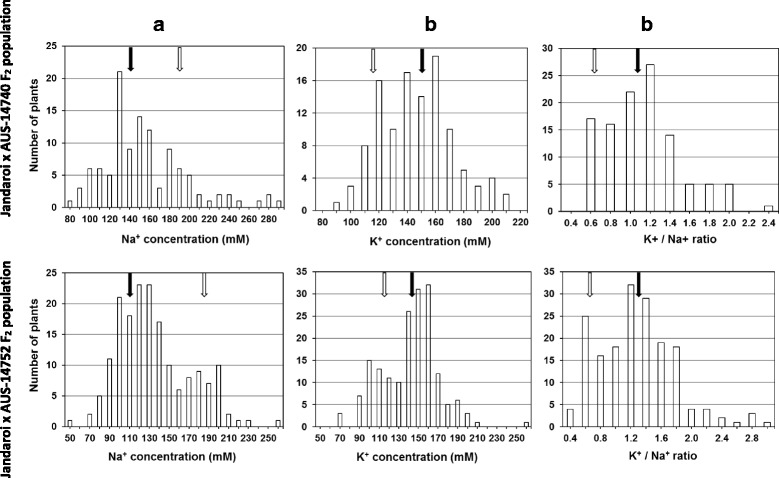
Distribution of third leaf Na^+^ and third leaf K^+^ concentration and the K^+^/Na^+^ ratio in F_2_ progeny of two crosses of durum wheat - Jandaroi × AUS-14740 (112 plants) and Jandaroi × AUS-14752 (176 plants). Bars represent number of plants for: (**a**) Third leaf Na^+^ concentration, (**b**) Third leaf K^+^ concentration and (**c**) K^+^/Na^+^ ratio. Black arrows indicate means for paternal parents, AUS-14740 and AUS-14752 (*n* = 10), and clear arrows indicate means for maternal parent, cv. Jandaroi (n = 10). Salt stress (100 mM NaCl) was gradually applied at the time of third leaf emergence. Na^+^ and K^+^ concentrations were measured in the fully expanded third leaf, 10 d after beginning of NaCl application

For both the Jandaroi × AUS-14740 and Jandaroi × AUS-14752 F_2_ populations, the distribution of all three traits studied (Na^+^ and K^+^ concentration in the third leaf and the K^+^/Na^+^ ratio) was bimodal (Fig. 1). Despite the differences between the two F_2_ populations, the trend for segregation was close to the 3:1 ratio of simple Mendelian genetics. The data presented indicates that the traits are closely linked, those lines with low leaf Na^+^ have high leaf K^+^ and a higher K^+^/Na^+^ ratios. This suggests a monogenic type of segregation for each of the three traits – third leaf Na^+^, third leaf K^+^ concentration, and the K^+^/Na^+^ ratio – in both populations studied. The segregation ratios observed in each of the three traits for both populations, with approximately three quarters of the lines studied having a similar phenotype to the landraces, indicate that the dominant alleles of the major genes originated from the two Afghani landraces (AUS-14740 and AUS-14752), Jandaroi contributing to the recessive alleles.

To estimate the genetic contribution to the phenotypic variation of the traits, the broad-sense heritability of Na^+^ and K^+^ concentration in the third leaf as well as the K^+^/Na^+^ ratio in both populations were calculated. The heritability of Na^+^ concentration in the third leaf for the F_2_ populations was particularly high and accounted for 0.89 and 0.78 of the variation for the Jandaroi × AUS-14740 and Jandaroi × AUS-14752 populations, respectively. The heritability of the K^+^/Na^+^ ratio was also estimated to be high in both populations, whereas the heritability of K^+^ concentration in the third leaf was moderate in the F_2_ population of the Jandaroi × AUS-14740 cross (0.41) and low in the F_2_ population Jandaroi × AUS-14752 (0.11) (Table 1).

**Table 1:** Analyses of parental lines and progeny in two F_2_ segregating populations. Concentration of third leaf Na^+^ and third leaf K^+^, and K^+^/Na^+^ ratio in F_2_ populations and parental forms (Jandaroi, AUS-14740 and AUS-14752) were measured and heritability values (*h* ^2^) were calculated after 10 d of salt stress (100 mM NaCl)

Population		Na^+^ (mM)	K^+^ (mM)	K^+^/Na^+^ ratio
Jandaroi × AUS-14740				
Parents	Jandaroi	168.5 ± 7.2	119.1 ± 6.5	0.70 ± 0.04
	AUS-14740	137.7 ± 8.9	147.4 ± 5.9	1.10 ± 0.07
F_2_ progeny	Mean	149.7 ± 3.9	141.2 ± 2.5	1.01 ± 0.04
	Minimum	79.8	83.8	0.45
	Maximum	232.7	209.2	2.25
Heritability (*h* ^*2*^)		0.89	0.41	0.79
Jandaroi × AUS-14752				
Parents	Jandaroi	185.7 ± 5.9	116.9 ± 10.6	0.64 ± 0.07
	AUS-14752	123.2 ± 14.8	140.2 ± 9.0	1.26 ± 0.14
F_2_ progeny	Mean	129.8 ± 2.7	138.0 ± 2.4	1.20 ± 0.04
	Minimum	49.3	60.4	0.26
	Maximum	266.1	230.0	2.92
Heritability (*h* ^*2*^)		0.78	0.11	0.66

### Bulk segregant analysis (BSA)

Application of the 9 K Infinium SNP genotyping assay to the two plant bulks with lowest and highest third leaf Na^+^ concentration indicated 17 markers that showed an association with third leaf Na^+^ concentration. The BSA revealed three significant QTL on the long arms of chromosomes 3B, 4B and 7A and two much smaller and insignificant QTLs on chromosome 1A. Five SNP markers in the genetic regions of the significant QTLs were selected and used for marker regression analysis (MRA) of the entire F_2_ populations (Additional file 1). These markers were successfully amplified using high resolution melting point technology and their alleles were shown to have clear melting curves similar to those for durum wheat. A marker*, Xm564,* on the chromosome 4B was found to have an association with the third leaf Na^+^ concentration with the favourable allele from AUS-14740. Also, *Xm5511* marker on the chromosome 3B showed a strong association with third leaf Na^+^ concentration. For this QTL the favourable allele was derived from Jandaroi. The two QTL on chromosomes 3B and 4B are significant with LOD score > 3 and each QTL explains 18% of the phenotypic variability for third leaf Na^+^ concentration (Table 2A).

**Table 2:** Association analysis between SNP markers and the three traits studied. Na^+^ and K^+^ concentration in third leaf, and K^+^/Na^+^ ratio were analysed in the F_2_ cross Jandaroi × AUS-14740. A LOD score of the association, the percentage of phenotypic trait variability and origin of the favourable allele were identified by marker regression analysis (MRA) in QTX MapManager

Chromosome	Marker	LOD score	Phenotypic variability (%)	Origin of favorable allele
(A) Na^+^ concentration in third leaf				
1A	*Xm2584*	0.6	3	Jandaroi
3B	*Xm5511*	3.4*	18*	Jandaroi
4B	*Xm6828*	0.3	2	AUS-14740
4B	*Xm564*	3.4*	18*	AUS-14740
(B) K^+^ concentration in third leaf				
3B	*Xm5511*	0.3	2	AUS-14740
4B	*Xm6828*	0.6	4	AUS-14740
4B	*Xm564*	3.9*	20*	AUS-14740
7A	*Xm3054*	0.8	4	AUS-14740
(C) Ratio K^+^/Na^+^				
1A	*Xm2584*	0.3	1.3	AUS-14740
3B	*Xm5511*	1.1	6	Jandaroi
4B	*Xm6828*	0.9	6	AUS-14740
4B	*Xm564*	5.6*	27*	AUS-14740
7A	*Xm3054*	1.5	7	AUS-14740

*Xm564* also showed a strong association with third leaf K^+^ concentration and the ratio of K^+^/Na^+^, with LOD scores of 3.90 and 5.58 respectively. This locus accounted for 20% of the variation in third leaf K^+^ concentration and 27% of the K^+^/Na^+^ ratio, but *Xm5511* showed no association with either trait (Table 2B and C).

## Discussion

Genetic loci for Na^+^ exclusion and K^+^/Na^+^ ratio were identified in a durum mapping population, produced by a cross between the elite Australian cultivar, Jandaroi and an Afghani exotic durum landrace, AUS-14740, which accumulates in leaves half the Na^+^ concentration compared to other durum wheats.

In the current study, the distributions of all traits (third leaf Na^+^ concentration, third leaf K^+^ concentration, and the K^+^/Na^+^ ratio) in both populations was suggestive of a bimodal indicating a possible monogenic segregation. These findings suggest that single major genes are associated with Na^+^ exclusion in both the Afghani durum landraces studied. Heritability for the traits studied was high in both populations, with the exception of third leaf K^+^ concentration. A moderate to high heritability for shoot Na^+^ concentration has previously been estimated in two F_2_ populations of durum wheat [19]; two major QTL, *Nax1* for *HKT1;4* and *Nax2* for *HKT1;5*, had been then identified in the same populations [13, 15]. Markers flanking these putative QTLs were used to develop salt tolerant durum wheat cultivars [17].

In order to define the genetic regions and identify potentially useful markers associated with third leaf Na^+^ concentration in the durum wheat of Afghani landraces, bulked segregant analysis (BSA) was conducted using SNP markers in the F_2_ population originating from the cross Jandaroi × AUS-14740.

In the Jandaroi × AUS-14740 F_2_ population, two SNP markers were found to be strongly associated with Na^+^ concentration in the leaves. The first marker, *Xm5511*, located on the long arm of chromosome 3B, was associated only with third leaf Na^+^ concentration and with neither third leaf K^+^ concentration nor the K^+^ /Na^+^ ratio. In contrast, a second marker, *Xm564*, was identified in the distal region of the long arm of chromosome 4B, and found to have a strong association with all three traits studied (Table 2).

Other QTL associated with these traits have been detected in other studies. The *Kna1* locus was mapped to the distal region of chromosome 4DL in bread wheat [20] and this locus is associated with the selective accumulation of K^+^ over Na^+^ in the shoot. In durum wheat, the *Nax2* locus was mapped to a region of the long arm of the chromosome 5A and this is associated with Na^+^ concentration and the ratio of K^+^/Na^+^ in the leaf blade [19]. It was found that *Nax2* encoded a similar gene as *Kna1* [15]. In rice (*Oryza sativa*), a QTL for shoot K^+^ content was mapped onto chromosome 1 in an F_2_ population derived from a cross between a salt tolerant indica (Nona Bokra) and an intolerant elite Japonica variety (Koshihikari). This QTL was named *SKC1* [21]. Low Na^+^ concentration in the leaves, improved selection of K^+^ over Na^+^ transported from roots to shoots, and a high ratio of K^+^/Na^+^ in the leaves are common phenotypic characteristics between *Nax2*, *Kna1* and *SKC1* [15]. As mentioned above, these characteristics were seen in the F_2_ population derived from Jandaroi and AUS-14740. A candidate gene of the *SKC1* locus is *OsHKT1;5*, which reduces the amount of Na^+^ transported from the root to the shoot [21]. An *HKT*-like gene was also cloned in *Kna1* and *Nax2*, and *HKT1;5* is a candidate for both loci; named as *TaHKT1;5-D* and *TmHKT1;5-A*, respectively [15]. *Kna1* is found in bread wheat (*T. aestivum* L.) whereas *Nax2* was derived from *T. monococcum*. It is possible that the *Xm564* locus identified in this study corresponds to the *Kna1* and *Nax2* loci and the candidate gene for the locus on the long arm of chromosome 4B is an *HKT*-like gene. Huang, et al. [22] found four bands of *HKT1;5*-like genes in bread wheat using a *HKT1;5* probe in Southern blots. Three hybridisation bands were located on the long arm of chromosome 4B and one band on the long arm of chromosome 4D. The *HKT1;5* gene has only been found in bread wheat and *T. monococcum*, and has not been identified in durum wheat [17]. The results suggest that Na^+^ exclusion in AUS-14740 may be due to the gene *HKT1;5*.

**Fig. 2:**
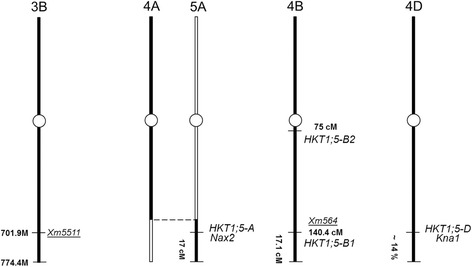
Mapping of two SNP markers, Xm5511 and Xm564, on chromosomes 3B and 4B and comparison with known locations of HKT1;5 genes in wheat. Both homoeologous genes in genomes A and D have similar genetic locations along the corresponding chromosomes. HKT1;5-A (Nax2) has been mapped onto the distal part of chromosome 5A with an estimated genetic distance of about 17 cM from the end of chromosome 5AL [15]. Part of chromosome 5AL originated from part of chromosome 4AL due to an ancient reciprocal translocation between the distal ends of chromosomes 4AL and 5AL [23]. HKT1;5-D (Kna1) has been identified in the genetic region about 14% from the end of chromosome 4DL [20]. Two genes; HKT1;5-B1 and HKT1;5-B2 on chromosome 4BL. HKT1;5-B1 is very close to the locus of Xm564 which is 17 cM from the end of chromosome 4BL, HKT1;5-B2 sits closer to the centromere on this chromosome

The locations of both markers on chromosomes 3BL and 4BL identified in the current study and known *HKT1;5* genes on chromosome 5AL in *T. monococcum* and on chromosome 4DL in bread wheat are shown in Fig. 2. Both homoeologous genes in the A and D genomes have similar genetic locations along the corresponding chromosomes. *HKT1;5-A* (*Nax2*) has been mapped to the distal part of chromosome 5A, with an estimated genetic distance of about 17 cM from the end of chromosome 5AL [15]. Part of chromosome 5AL originated from part of chromosome 4AL due to an ancient reciprocal translocation between the distal ends of chromosomes 4AL and 5AL [23]. *HKT1;5-D* (*Kna1*) has been identified in the genetic region about 14% from the end of chromosome 4DL [20].

We used the IWGSC website (http://pgsb.helmholtz-muenchen.de/plant/wheat//iwgsc/index.jsp) and the Gramene website to blast sequences from the Infinium design, where each SNP marker (used in our study) was developed, against the sequence of wheat chromosome 4B. We identified *Xm564* markers on the distal part of chromosome 4BL in wheat contig 7,009,377, along with another marker, wsp_BG604404B. The position of *Xm564* was 140.39 cM, out of a total length of 157.59 cM for the chromosome 4B genetic map, the marker being located about 17.1 cM from the end of chromosome 4B (Fig. 2). The *HKT1;5-B1* like gene is also located on the wheat contig 7,009,377 and is in a very strong candidate for gene underlying this locus. This hypothesis, however, needs to be tested by looking for variation in or around the target gene by genotyping *HKT1;5-B1* in the F_2_ progeny from the cross Jandaroi × AUS-14740. In future, sequencing of the *HKT1;5-B1* gene from AUS-14740 and Jandaroi would determine whether there were differences in the protein encoded by the two alleles. If there were differences, heterologous expression of the two alleles of *HKT1;5-B1* in yeast and *Xenopus* oocytes would investigate whether differences in coding sequence resulted in altered function of the protein and its affinity for the Na^+^ ion. In addition, quantitative RT-PCR would determine whether the differences in the Na^+^ phenotype observed between AUS-14740 and Jandaroi is due to differences in spatial and/or temporal expression the *HKT1;5-B1* gene between the two accessions. It would also be important to make a separate study of the Jandaroi × AUS-14752 segregating population, as the two exotic Afghani landraces may contain different alleles of *HKT1;5-B1* gene.

Using genetic similarity between rice and wheat genes, we were able to localise the location of a second HKT1;5 gene on chromosome BL, *HKT1;5-B2*. This gene, however, is found in the near-centromeric region of the chromosome 4BL and is unlikely to be responsible for the phenotype linked to marker Xm564 (Fig. 2).

The role played by other possible genes remains unclear. SNP marker *Xm5511* was located on the chromosome 3BL, and it was found to make a similar, high, contribution to Na^+^ concentration in the third leaf (Table 2). We mapped the marker *Xm5511* to the distal part of chromosome 3BL, with a physical location of about 701.9 Mbp, out of the 774.4 Mbp total length of the chromosome 3B physical map (Fig. 2). However, the favourable allele here originates from the Jandaroi parent rather than the Afghani accession, indicating that Jandaroi possesses other genes that are directly involved in the mechanism of Na^+^ exclusion.

It should be noted that Jandaroi was developed as the elite durum wheat cultivar adapted to the growth conditions prevalent in Australia; nevertheless, like other existing cultivated durum germplasms, Jandaroi, accumulates very high leaf Na^+^ concentrations, when compared to bread wheat [6, 8, 18]. This is typically because durum wheat do not contain an active native *HKT* gene involved in excluding Na^+^ from the shoot [6, 15, 16]. To date the only durum wheat with significant reductions in shoot Na^+^ have had *HKT* genes have had them introduced through selective processes, such as the incorporation of the *Kna1* locus from the D genome of bread wheat [7, 15] or the *Nax1* and *Nax2* locus from *T. monococcum* [15, 17]. At this stage, there is no published information available that an *HKT*-like gene or any other potential candidate genes are located in the relevant genetic region of the chromosome 3BL. Therefore, a Jandaroi allele for Na^+^ exclusion with this region is unique. Consequently, further study is required to shed light on this particular locus, which seems to be very different from those in durum landraces.

## Conclusion

Two loci affecting shoot Na^+^ concentration were identified in the F_2_ segregating population, Jandaroi x AUS-14740, located on the long arms of chromosomes 4B and 3B. The loci need to be further fine mapped and candidate genes identified using SNP markers. This would facilitate the construction of a fine linkage map of the corresponding genetic regions. The *HKT1;5-B1* gene identified in this study on the long arm of chromosome 4B requires further analysis as the most suitable candidate gene in both segregating populations originating from exotic Afghani landraces. Expression profiling of the Jandaroi and AUS-14740 alleles will indicate whether the difference in Na^+^ phenotype is due to differences in expression between the two accessions, while heterologous expression of the *HKT1;5-B1* gene in yeast and *Xenopus* oocytes will enable characterisation of the transport properties of the protein. Marker assisted selection and backcrossing would facilitate the introduction of the beneficial allele of *HKT1;5-B1* from AUS-14740 into Jandaroi, similar to the approaches used to introduce the *Nax2* [17] and *Kna1* [7] alleles into durum. Saline field trials would then determine the effect it has on yield. The relevant genetic region on the chromosome 3BL needs to be studied more extensively with a range of molecular markers in order to identify other candidate genes in the genetic region originating from cultivar Jandaroi.

## Methods

### Production of lines for mapping

Seeds of two durum landraces originating from Afghanistan, Gandum Siahloshe Zamistani Aubi (AUS-14740) and Gandum Kofari (AUS-14752), and the cultivar Jandaroi were obtained from the Australian Winter Cereal Collection, Tamworth, NSW, Australia. Jandaroi is an Australian elite durum cultivar adapted to central and southern Queensland and some parts of New South Wales, Western Australia and South Australia. AUS-14740 and AUS-14752 were selected for this study as they had been previously described as having good Na^+^ exclusion [18, 24]. Plants of Jandaroi were emasculated and pollinated by pollen collected from plants of either AUS-14740 or AUS-14752. Hybrid F_1_ plants were self-pollinated and two corresponding F_2_ populations were produced. These F_2_ populations comprised 112 plants for Jandaroi × AUS-14740 and 176 plants for Jandaroi × AUS-14752, and were used for genetic analysis of Na^+^ concentration in the leaves.

### Growth conditions

Seeds of the parents and two F_2_ populations were placed on moist filter paper in Petri dishes (10 seeds per dish), incubated at 4 °C for 24 h and then placed at room temperature for 4 days to germinate. Uniform-sized seedlings were transplanted into a supported hydroponics system [25], as modified by Shavrukov et al. [24, 26]. Each population was grown in a separate hydroponics trolley, which had two tubs, each divided into ten rows and 16 columns. Ten days after transplanting, when the third leaf emerged, NaCl was gradually applied to the growth solution in daily increments of 25 mM NaCl until 100 mM NaCl was achieved. Supplemental calcium was added to give a Na^+^:Ca^2+^ of 30:1. The experiments were carried out in greenhouses on the Waite Campus, University of Adelaide, South Australia, and the temperature was adjusted to between 22 and 24 °C during the day and 14 to 18 °C at night. The light regime was a cycle of 14 h daylight/10 h dark with the use of additional lighting (400 W/lamp) when the level of light outside varied.

### Experimental design and statistical analysis

A spatial design comprising rows and columns within a tub was used to assess Na^+^ and K^+^ concentration in the third leaf of F_2_ plants. Each parent was randomly replicated ten times per trolley. Replicates of parents were used to estimate experimental error and environmental effects, applying restricted maximum likelihood (REML) using GenStat software [27] with a mixed model to estimate the broad-sense heritability of phenotypic traits [28]. To estimate the heritability for each trait, two models were developed for fixed and random terms. The various genotypes (Jandaroi, AUS-14470, AUS-14752, and F_2_ plants) were treated in the fixed model as imposed factors. In the random model, the F_2_ plants, rows, columns, and tubs were considered random factors. Broad-sense heritability was calculated as a ratio of genetic component variance in F_2_ plants to total component variance. Best linear unbiased estimates (BLUEs) were then generated by incorporating the data of individual F_2_ plants into the fixed model and rows, columns, and tubs into the random model [29], the BLUEs then used for the marker regression analysis.

### Measurement of Na ^+^ and K^+^ concentration in the third fully expanded leaf

Plants of the two F_2_ populations and the parents were grown for 10 days after the application of NaCl and the fully expanded blade of the third leaf was harvested. The fresh weight of the leaf samples were determined before the tissue was oven-dried at 65 °C for 48 h and dry weights recorded. The concentrations of Na^+^ and K^+^ in the third leaf were measured using a flame photometer as described previously [18, 24].

### DNA extraction

DNA extraction was performed using a midi-prep method [30] for bulk segregant analysis of the F_2_ plants. In this method, 2 g of leaf sample from leaves below the flag leaf was used. Freeze-dried DNA extraction was performed for the entire F_2_ population [31] using young leaves of seedlings.

### Bulk segregant analysis (BSA) and genotyping

BSA was conducted on the F_2_ cross, Jandaroi × AUS-14740. Two bulks were generated according to the lowest and highest Na^+^ concentration in the third leaf. DNA samples were pooled from the 20 F_2_ plants with the lowest (Bulk 1) and 20 F_2_ plants with the highest third leaf Na^+^ concentration (Bulk 2).

DNA from Bulk 1, Bulk 2, AUS-14740 and Jandaroi were run on a 9 K Infinium SNP and analysed on a BeadStation and iScan instruments (Illumina) [32]. All Gene-Call data were manually checked and positive hits were recorded when SNPs were polymorphic between Jandaroi and AUS-14740. The SNPs were assessed for tight clustering: Bulk 1 for AUS-14740 and Bulk 2 for Jandaroi. The genotyping analysis was carried out at AgriBiosciences Centre, Victoria, Australia.

After the putative SNP markers were identified from the BSA, the sequence of each marker obtained from the Infinium wheat SNP platform (http://wheat.pw.usda.gov/ggpages/9K_assay_available.html) was used to generate the forward and the reverse primers, using Primer 3 software (http://frodo.wi.mit.edu/). All primers were designed to generate amplicons 100 to 300 bp long. The annealing temperature for all primers was 60 °C. The GC content (CG%) of the designed primers ranged between 40 and 60%. The possibility of dimerisation and hairpins on the designed primers were tested using NetPrimer software (http://www.premierbiosoft.com), and those with a ∆G of more than −5 kcal/mol were re-designed. Primers were ordered from GeneWorks (http://www.geneworks.com.au) and validated on the two parents, F_1_ hybrids and 12 F_2_ lines. The fragments were amplified with designed primers and genotyped using High Resolution Melting Point technology [33].

### Marker regression analysis (MRA)

To analyse the association between SNP markers and phenotype (concentration of Na^+^ and K^+^ in the third leaf, and the K^+^/Na^+^ ratio) in the entire F_2_ population, an MRA was conducted using QTX software, MapManager [34]. The report from the analysis lists the chromosome name, the locus (marker) name, the likelihood ratio statistic (LRS), the percentage phenotypic variation explained by the locus and the parental allele. The LRS value was converted to a LOD score using the formula LOD score = LRS/4.61 [35]**.** The parental allele was estimated as positive if it tended to increase the trait value; similarly, if the presence of the parental allele tended to decrease the trait value, then it was estimated as negative.

## Additional file

Additional file 1: Data for seven SNP markers used in this research. Details regarding the markers used in this study, which contains their sequence information and the locus ID of the gene the marker is within. (DOCX 14 kb)

## Abbreviations

BSA: Bulked segregant analysis
HKT: High affinity potassium transporter
IWGSC: International wheat genome sequencing consortium
K^+^: Potassium
LOD: Log of odds
MRA: Marker regression analysis
Na^+^: Sodium ion
QTL: Quantitative trait locus
SNP: Single nucleotide polymorphism
