# Supplementary material for: Genetics of Na+ exclusion and salinity tolerance in Afghani durum wheat landraces
Source: BMC Plant Biol. 2017 Nov 21;17:209. doi: 10.1186/s12870-017-1164-6 (PMC5697363; doi:10.1186/s12870-017-1164-6)
Supplement: Additional file 1: — Data for seven SNP markers used in this research. Details regarding the markers used in this study, which contains their sequence information and the locus ID of the gene the marker is within. (DOCX 14 kb) [file 12870_2017_1164_MOESM1_ESM.docx]

## Additional file 1

**Data for seven SNP markers used in this research**

***Xm7512***

***Chromosome*** 3B: 575,863,598-575,863,752

***Gene:*** LOC542932, Traes_3B_C0F416702, F1-ATPase

***Ortholog:*** No

Sequence: GCCAAAATTGCTGTAGCCAATTTTGTCGCTATAGTTTTGGGGTGGGTCAAGGAATGATCGCATCATTGAATAATTTGAGATTTGACAGCACGCTTGCTCG[A/G]ACTTATCATGTGTAACATTGAGAAAG

***Xm5511***

***Chromosome*** 3B: 701,901,110-701,901,330

***Gene:*** TRAES3BF010200060CFD_g

***Ortholog:*** BGIOSGA034258, Putative uncharacterized protein [Source: UniProtKB/TrEMBL; acc: B8BJS3].

***Sequence:*** AGATGACGCGTGTTCCAGAGGTTCRTGTCTGAATGCGGCATAGTGGTTCTTTTTTCTGCAGTGGCATTCTGGTATGGTGCATCCATGTCATTCTAGATTT[A/C]ATAATTCTGGCATGTTACAATTCTTAAGACACCATTTGCGAGTCTTGTACTTTTGTATCGTGATACGGTTTCTGTTTTGAAAATGCCGGTGTTCATTGCC

***Xm6828***

***Chromosome*** 4B: 284,858,090-284,862,842

***Gene:*** Traes_4BL_031666EE1

***Ortholog:*** BGIOSGA016180, Putative uncharacterized protein [Source: UniProtKB/TrEMBL; acc: B8AST5].

Sequence: CATCCATGCACCCTCTCCCTCTATACTTTGGCAATTTCATAGAGGCATGGCATTTTACATTGGCAAAGACTGAACTCCTATATGTAGCTTAAGCTTGTAT[T/C]ACTAGGCTTGTAATCTGTATTATCAACGGTAAGATACAGTTAATTAAATCATATTTACAAADWAGTC

***Xm564***

***Chromosome*** 4B: CSS_4BL_scaff_7009377: 2,594-5,865

***Gene:*** Traes_4BL_0E93AFC51, GroES-like zinc-binding alcohol dehydrogenase family protein [Source:Projected from *Arabidopsis thaliana* (AT5G61510) TAIR;Acc:AT5G61510].

***Ortholog:*** OS03G0101600, Os03g0101600 protein [Source: UniProtKB/TrEMBL; acc: Q0DW19].

Sequence: AGGAACCCTGAGTGGAAACCCTCTAGCTATGACTGCCGGAATCCACACTCTCAAGCGTCTGATGGAGCCTGGCACCTATGAATACTTAGACAAGGTCACC[A/G]GTGAACTTGTCCAGGGTATATTGGATGCGGGTGCTAAAACAGGGCACGAGATGTGTGGAGGACACATCAGAGGCATGTTCGGATTCTTCTTCGCAGGTGG

***Xm3054***

***Chromosome*** 7A: CSS_7AS_scaff_4251143: 4,771-7,800

***Gene:*** Traes_7AS_9C283FE66

***Ortholog***: OS08G0532200, GSA, Glutamate-1-semialdehyde 2,1-aminomutase, chloroplastic [Source: UniProtKB/Swiss-Prot; acc: Q6YZE2].

Sequence: AGGAACCCTGAGTGGAAACCCTCTAGCTATGACTGCCGGAATCCACACTCTCAAGCGTCTGATGGAGCCTGGCACCTATGAATACTTAGACAAGGTCACC[A/G]GTGAACTTGTCCAGGGTATATTGGATGCGGGTGCTAAAACAGGGCACGAGATGTGTGGAGGACACATCAGAGGCATGTTCGGATTCTTCTTCGCAGGTGG
